# Incidence of Liver Resection Following the Introduction of Caseload Requirements for Liver Surgery in Switzerland

**DOI:** 10.1007/s00268-022-06509-w

**Published:** 2022-03-16

**Authors:** Fabian Haak, Savas Soysal, Elisabeth Deutschmann, Giusi Moffa, Heiner C. Bucher, Max Kaech, Christoph Kettelhack, Otto Kollmar, Marco von Strauss und Torney

**Affiliations:** 1 Clarunis, Department of Visceral Surgery, University Centre for Gastrointestinal and Liver Diseases, St. Clara Hospital and University Hospital Basel, Spitalstrasse 21, 4031 Basel, Switzerland; 2 Basel Institute for Clinical Epidemiology and Biostatistics, Department of Clinical Research, University Hospital Basel, and University of Basel, Basel, Switzerland; 3 Department of Surgery, Bürgerspital Solothurn, Solothurn, Switzerland; 4 University of Basel, Basel, Switzerland

## Abstract

**Background:**

Centralization of care is an established concept in complex visceral surgery. Switzerland introduced case load requirements (CR) in 2013 in five areas of cancer surgery. The current study investigates the effects of CR on indication and mortality in liver surgery.

**Methods:**

This is a retrospective analysis of a complete national in-hospital data set including all admissions between January 1, 2005, and December 31, 2015. Primary outcome variables were the incidence proportion and the 60-day in-hospital mortality of liver resections. Incidence proportion was calculated as the overall yearly number of liver resections performed in relation to the population living in Switzerland before and after the introduction of CR.

**Results:**

Our analysis shows an increase number of liver resections compared to the period before introduction of CR from 2005–2012 (4.67 resections/100,000) to 2013–2015 (5.32 resections/100,000) after CR introduction. Age-adjusted incidence proportion increased by 14% (OR 1.14 95 CI [1.07–1.22]). National in-hospital mortality remained stable before and after CR (4.1 vs 3.7%), but increased in high-volume institutions (3.6 vs 5.6%). The number of hospitals performing liver resections decreased after the introduction of CR from 86 to 43. Half of the resections were performed in institutions reaching the stipulated numbers (53% before vs 49% after introduction of CR). After implementation of CR, patients undergoing liver surgery had more comorbidities (88 vs 92%).

**Conclusion:**

The introduction of CR for liver surgery in Switzerland in 2013 was accompanied by an increase in operative volume with limited effects on centralization of care.

## Introduction

In the era of cost constrained health care due to demographic changes and limited budgets, optimizing quality of care at acceptable costs is an unifying target. However, a precise definition of quality of care is amenable to different perspectives of different stakeholders. Providers such as hospitals, national medical societies, individual physicians, politicians and finally patients play a role in this complex process and at times have conflicting interests.

Driven by these complex relationships, national healthcare systems have taken different approaches to centralize health care in highly specialized medicine where either the outcome/volume relationship has previously been proven, or high costs made centralized services necessary [1]. However, only 13 European countries have introduced minimal number requirements for certain surgical procedures, showing that while there seems to be convincing evidence of the effect of volume-based centralization of specialized health care, only a limited degree of implementation has taken place [2].

The concept of centralization in surgery is not new. In 1979, Luft et al. described the relationship between case load and decreasing mortality [3]. Several other research groups also addressed this question within subsequent years [4–7]. The definite causative factors for the inverse relationship between postoperative mortality and higher surgical volume are unknown but likely multifactorial. It is clear that the procedure itself plays an important role, favoring complex procedures for centralization to reduce mortality [4]. A superiority of high volume centers due to better knowledge about indications, procedural technique and postoperative care could have an impact as well [8, 9]. The “rescue phenomenon,” or the ability to recognize and manage postoperative events, thereby preventing the development of more severe problems, seems to be one if not the most important factor for the superior outcomes of large centers. [10].

The introduction of CR for esophagectomy, pancreatectomy, major liver resection, rectal cancer surgery and bariatric surgery in 2013 marked the beginning of centralization of surgical procedures in Switzerland, with the goal to intensify inter-hospital competition specialized surgery and to foster centralization of care. However, the case load requirement up to present was not endorsed with any form of sanctions for those hospitals not fulfilling the CR criteria. This offers an unique opportunity to study the trends that might influence clinical decision making in the light of these competing interests and their influence on outcome.

Actually, 17 centers in Switzerland are currently licensed to perform hepatic surgery. Our study investigates whether the introduction of caseload requirements in Switzerland has led to a change in overall operative volume and mortality after major liver resections.

## Methods

We used anonymized data from the Federal Statistical Office (FSO) Office inpatient registry which collects data according to a mandatory and standardized data collection form [11]. The database includes administrative and demographic patient data (month year and mode of admission, age in 5-year intervals and gender), as well as main and secondary diagnoses and treatment codes for main and secondary treatments as well as a limited set of outcome variables including in-hospital mortality.

The sample size was given by the number of patients admitted to the hospitals in Switzerland for major liver resections from January 1, 2005, until December 31, 2015. Resections were defined by Swiss procedure codes (CHOP) (Appendix Table 4).

Primary outcome was the incidence proportion of liver resection partial (hepatectomy or lobectomy, for details see below), while secondary outcome was the 60-day in-hospital mortality in patients with liver resection. The incidence proportion was calculated as the ratio of the number of total resections divided by the number of people living in Switzerland in the given year [12]. The adjusted rate, including the lower and upper confidence intervals, considers the changing age distribution of the underlying populations over the years. A change of the incidence proportion of >5% was defined and considered a clinically relevant increase prior to conducting the analyses.

The 60-day in-hospital mortality was calculated as the number of patients who had died in hospital (either at their index admission or at consecutive admissions within 2 months afterward) divided by the number of patients with liver resections.

Based on secondary diagnoses, the Charlson Comorbidity Index (CCI) was calculated as a measure for the burden of comorbidity [13]. The definition of hospital class differentiates between medical centers (defined by an average hospital admission caseload of ≥ 7500 patients per year) and regional hospitals (defined by an average hospital admission caseload of <7500 patients per year). The minimal caseload requirement for liver surgery according to Swiss health authorities has been set at 20 (12) anatomic hepatic resections per year [14]. Hospital expertise was classified as clearly above caseload requirement (>20% of stipulated volume, >24 resections), around caseload requirement (±20% of the stipulated volume, 16–24 resections) or clearly below (<20% of the stipulated volume, <16 resections. Mortality was analyzed according hospital expertise and compared before and after the introduction of caseload requirement.

The recording of liver resections in Switzerland has evolved during the study period: Until 2008, major liver resections (according to Swiss Health authorities, Appendix Table 4) were classified either as partial hepatectomy or lobectomy. From 2009 onwards, lobectomies were subclassified. From 2011 onwards, subclassifications for partial hepatectomies were documented. Since this could have influenced overall caseload, we performed a sensitivity analysis accounting for this sub-classification.

Qualitative data were summarized in contingency tables (including percentages) presented overall and by condition/procedure. Quantitative data were summarized by appropriate descriptive statistics including, e.g., the number of observations, arithmetic mean and standard deviation or median and interquartile range (IQR), minimum and maximum, number of missing observations. Continuous variables were log-transformed for the analysis; the geometric mean was reported, together with its coefficient of variation. The relative risk (RR) for liver surgery before / after caseload introduction was calculated. Age-adjusted RR was calculated as the ratio of risks of having a resection before vs. after introduction of caseload. Data analysis was performed using R Version 3.4.1 (R Foundation for Statistical Computing, Vienna, Austria) and SAS 9.4.

## Results

For the entire observation period from 2005 to 2015, 4939 liver resections in 4597 patients were reported, with 310 patients having multiple liver resections. The patient population of liver resection patients before and after caseload introduction was homogenous. Gender, nationality and type of health insurance coverage all remained within a two percent shift of the total population distribution. 54% of liver resections were performed in male patients. Most of the liver resections (94%) were performed electively. Before the introduction of caseload requirements, 88% of the patients had one or more relevant comorbidities (CCI > 0). This increased to 92% after the caseload requirements were introduced. After the introduction of CR, the percentage of patients treated in small institutions such as regional hospitals decreased from 8 to 2% (Table 1).

**Table 1:** Demographics liver resections

	Overall	Before implementation of caseload requirements (2005–2012)	After implementation of caseload requirements (2013–2015)
Gender			
Male (%)	2505 (54.5)	1704 (54.0)	801 (55.5)
Female (%)	2092 (45.5)	1449 (46.0)	643 (44.5)
Citizenship			
Swiss (%)	3676 (80.0)	2521 (80.0)	1155 (80.0)
Non-Swiss (%)	920 (20.0)	631 (20.0)	289 (20.0)
Type of health insurance coverage of patients			
Basic (%)	1625 (35.4)	1132 (35.9)	493 (34.1)
Supplementary, private (%)	2968 (64.6)	2017 (64.0)	951 (65.9)
Admission mode			
Elective (%)	4318 (93.9)	2956 (93.8)	1362 (94.3)
Emergency (%)	269 (5.9)	191 (6.1)	78 (5.4)
Unknown (%)	10 (0.2)	6 (0.2)	4 (0.3)
Patient comorbidities			
Charlson comorbidity Index > 0 (%)	4097 (89.1)	2770 (87.9)	1327 (91.9)
Hospital class			
Medical center (%)	4311 (93.8)	2895 (91.8)	1416 (98.1)
Regional hospital (%)	286 (6.2)	258 (8.2)	28 (1.9)

**Fig. 1:**
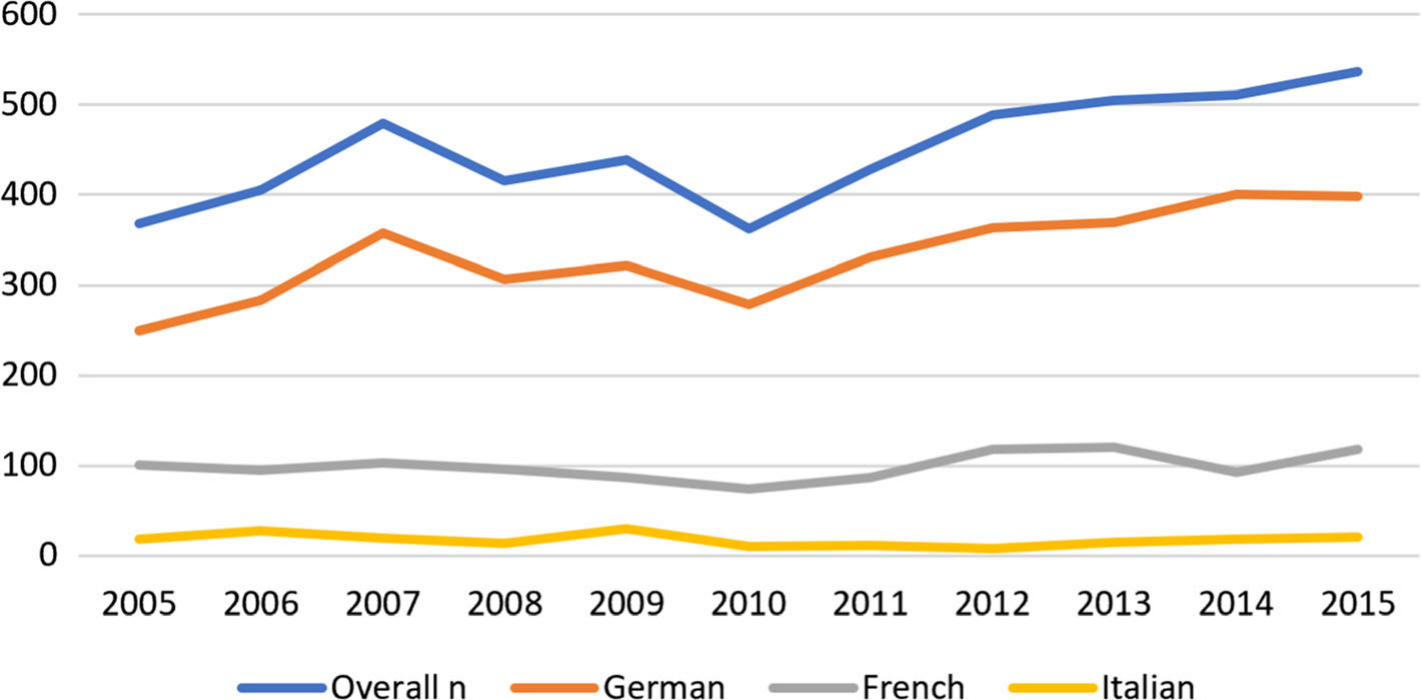
Distribution of resections among language regions

The unadjusted yearly incidence of liver resections in relation to the Swiss residential population (incidence proportion) increased from 4.67 resections/100,000 for the 2005–2012 period to 5.32 resections/100,000 for the 2013–2015 period (Table 2). The age-adjusted incidence proportion increased by 14% (Relative risk 1.14 (95 CI [1.07–1.22]) after introduction of caseload requirements. The distribution of resections per hospital in different language regions of Switzerland shows that the majority of resections were performed in the German speaking part of the country. Here the amount of hospitals being above the cutoff stayed the same while the number of hospitals below the cutoff decreased (Fig. 1).

**Table 2:** Yearly liver resections—incidence proportion per 100,000

Year	Resections (*n*)	Population CH (*n*)	Crude rate	Adjusted rate (95%)
2005	359	7,459,128	4.81	4.52 (3.95, 7.25)
2006	385	7,508,739	5.13	4.73 (4.18, 7.32)
2007	443	7,593,494	5.83	5.10 (4.54, 7.47)
2008	366	7,701,856	4.75	4.66 (4.14, 6.93)
2009	403	7,785,806	5.18	4.52 (4.01, 6.67)
2010	331	7,870,134	4.21	4.08 (3.57, 6.66)
2011	401	7,954,662	5.04	4.73 (4.18, 7.20)
2012	465	8,039,060	5.78	5.04 (4.50, 7.82)
2013	467	8,139,631	5.74	5.32 (4.75, 8.00)
2014	474	8,237,666	5.75	5.00 (4.45, 7.71)
2015	503	8,327,126	6.04	5.64 (5.08, 8.31)

The in-hospital mortality ranged from 9 deaths in 2005 (minimum) to 29 deaths in 2009 (maximum). The 60-day in-hospital mortality rate was 1.4% in 2008 (minimum) and 7.4% in 2009 (maximum). The all-cause mortality rate was 18% (16%–24%) before caseload introduction and 10% (6%–17%) after caseload introduction.

Before the introduction of caseload requirements, half of the resections (*n* = 1823 (53.82%)) were performed at the 6 institutions which already met the caseload requirements. The other half (*n* = 1564 (46.18%)) were performed in low volume institutions (80 in total), which at the time did not meet the caseload requirements.

Looking at mortality rates in relation to caseload, the results of hospitals performing liver resections were subdivided in institutions clearly below caseload (<20% of the stipulated volume), around caseload (±20% of the stipulated volume) and clearly above caseload (>20% of stipulated volume). Before caseload introduction, 77 hospitals performed resections while being clearly below caseload. The mortality in this group was 4%. Four hospitals performed resections while being close to the required caseload. The mortality in this group was 4.8%. Five hospitals performed resections while being clearly above caseload, and the mortality was 3.5% in this group. After caseload introduction in 2013, 43 of the remaining hospitals performing liver resections were clearly below caseload. The mortality in this group was 2.2%. Seven hospitals performed resections while being close to the caseload, and the mortality was 1.8%. Six hospitals performed resections while being clearly above caseload, with the mortality being 5.5% in this group after CR introduction (Table 3).

**Table 3:** Primary and secondary outcomes

	Before caseload introduction			After caseload introduction		
	Crude rate	Adjusted rate	Confidence interval	Crude rate	Adjusted rate	Confidence interval
Incidence proportion (number of resections)	5.1	4.7	4.5–5.0	5.8	5.3	5.0–6.2
In-hospital mortality (percent)	4.9%	4.9%	3.1%–10%	4.2%	3.9%	1.4%–11%
All-cause mortality (percent)	29%	18%	16%–24%	13%	10%	6%–17%
Hospital expertise*						
Number of resections in hospitals above CR requirements (%)	1823 (53.8%)			752 (48.5%)		
Number of resections in hospitals below CR requirements (%)	1564 (46.2%)			800 (51.6%)		

^*^Not adjusted for age

Seven hospitals passed from a lower experience class to a higher class after caseload introduction. Before caseload introduction, these hospitals had performed an average of 10 resections per year. After introduction of caseload requirements, they increased their average yearly resections to 22. However, at the same time mortality increased from 2.7% to 3.8% in this group.

## Discussion

Our data document a trend toward an increase in major liver resections which commenced already in the years prior to the CR implementation when the new rules were announced by Swiss health authorities and further rose after their introduction. Next to this, there is evidence that the intended centralization of liver resections failed at least until 2015. Instead, we could show that the introduction of caseload requirements was accompanied by an overall increase in resections by 14%, the majority of which are performed in hospitals not meeting the stipulated caseload cutoffs.

While growing evidence is available for an effect of centralization on outcome after pancreatectomy and esophagectomy, it is still scarce in the field of liver surgery [5, 15, 16]. Recent data from Germany show that mortality after major liver resection remains high [17, 18]. For example, mortality for right hemihepatectomy with bile duct reconstruction for malignancy was found to be as high as 30.8%. In concordance with to Switzerland, liver resections were not regulated in Germany until recently. Between 2010 and 2015, 1136 hospitals performed major liver resections. Outcomes were not reviewed by an independent administrative body unless the centers voluntarily sought certification by the German Association for General and Visceral Surgery or the German Cancer Society in a Comprehensive Cancer Network programme [19]. However, certification failure did not prevent the centers from performing liver surgery in the future. Multiple studies looking at the mortality exist also from other countries. They report significant lower rates for a variety of liver procedures [20–27].

Our study could not detect an effect of the introduction of case load requirements on in-hospital mortality. Compared to the above-mentioned recently published data from Germany, the overall mortality after liver surgery in Switzerland seems to have been highest 2009 at 7.4 percent, but the comparison is limited as caseload composition was different.

The growing proportion of patients with major live resections and higher CCI indicates that surgery is offered to frailer patients. It remains unclear if this reflects the evolution of surgical and anesthesiology technique or if it is an expression of institutions struggling for cases to reach the cutoffs. At least this increase was not accompanied by a consecutive rise in mortality.

Based on our data, it remains unclear if case distribution based on market principles and competition between centers to achieve centralization is effective. Most likely it did lead to broader indications in sicker patients, but with only a limited effect on the intended effect on centralization. This hypothesis is underlined by the found increase in the CCI in patients undergoing liver resection in the post-CR introduction area. Allocating surgical procedures to institutions based on predefined criteria such as size, available infrastructure and skills would possibly allow to achieve the postulated goal without the side effect of an increased overall caseload. Another alternative to competition-based allocation could be the use of objective measures like the risk-standardized mortality rate (RSMR) [28].

This analysis has several relevant limitations. The available baseline data did only cover the start of the introduction of caseload requirements in Switzerland from 2013 to 2015, during which many hospitals successfully appealed against the CR that were imposed on their institutions by the Swiss Health authorities and continued to deliver surgery treatments. Nevertheless, all providers knew that even during this transition, caseload would be the basis for future allocations and therefore stimulating the competition between institutions. The database does only include inpatient data; therefore, important and relevant information on oncological outcomes, out of hospital mortality and non-fatal surgical complications was not available. Likewise the registry does not include any information on intra- and postoperative complications from surgery. Probably most relevantly, we had no data of patients with conservative management, and therefore had to use an alternative denominator and could only analyze patients undergoing resections in relation to the overall population. In addition, lack of detailing of types of resections during the early period of the registry did not allow us to look into detailed outcomes according to the liver resection type over the entire study period. All these factors limit the generalizability of our findings. Nevertheless, due to the comprehensiveness of the database, including all liver resections performed in Switzerland in this 11 year period, we feel confident that some basic conclusions can be drawn.

## Conclusion

The competition-based introduction of CR for liver surgery in Switzerland in 2013 was accompanied by an increase in operative volume with only limited effect on the intended centralization of care.

## Appendix

See Table 4

**Table 4:** Operation codes (CHOP)

Code (CHOP)	Procedure
Z50	Liver resection
Z50.22	Partial hepatectomy
Z50.22.10	Partial hepatectomy, percutaneous partial hepatectomy (local excision, endocyst resection, wedge resection)
Z50.22.20	Partial hepatectomy, endocyst resection, open
Z50.22.21	Partial hepatectomy, endocyst resection, laparoscopic
Z50.22.40	Partial hepatectomy, segment resection
Z50.22.41	Partial hepatectomy, multiple segment resection, not adjoined
Z40.22.42	Partial hepatectomy, partial hepatectomy for transplantation
Z40.22.99	Partial hepatectomy, not otherwise specified
Z50.3	Liver lobectomy
Z50.3×.00	Liver lobectomy, not otherwise specified
Z50.3×.01	Lobectomy, hemihepatectomy, resections of several liver segments, resection for transplantation excluded
Z50.3×.11	Lobectomy, hemihepatectomy, resections of several liver segments, for transplantation

.
